# Supplementary material for: Partitioning of ribonucleoprotein complexes from the cellular actin cortex
Source: Sci Adv. 2022 Aug 19;8(33):eabj3236. doi: 10.1126/sciadv.abj3236 (PMC9390997; doi:10.1126/sciadv.abj3236)
Supplement: Supplementary file 1 — Text S1 and S2 Figs. S1 to S8 Tables S1 and S2 References [file sciadv.abj3236_sm.pdf]

Science Advances
AAAS

Supplementary Materials for

Partitioning of ribonucleoprotein complexes from the cellular actin cortex

Isaac Angert *et al.*

Corresponding author: Joachim D. Mueller, jochen@umn.edu

Sci. Adv. **8**, eabj3236 (2022)
DOI: 10.1126/sciadv.abj3236

This PDF file includes:

Text S1 and S2
Figs. S1 to S8
Tables S1 and S2
References

Supplemental Text 1: Derivation of Eq 1

Eq. 1 is derived by preserving the integrated area Λ under the z-scan intensity profile between the SSS^G model and the fitted S^G model. Since Λ and the axially integrated concentration A are directly proportional (Eq. 7), the concentration areas of both models (Fig. 4C and D) must match. In particular, the cortical concentration area $A_C^i = L_C^i \cdot c_C$ (Fig. 4C) has to be equal to the added extra area $A_x^i = \left(L_C^i - \Delta^i\right) c_{cyto}$ (Fig. 4D) at both the top (i = T) and bottom (i = B) of the cell. Thus, using $R_C = c_C / c_{\mathrm{cyto}}$ we get $\left(L_C^i - \Delta^i\right) c_{\mathrm{cyto}} = L_C^i R_C c_{\mathrm{cyto}}$, which is equivalent to Eq.1.

Supplemental Text 2: The axially averaged partition coefficient $\bar{R}_z$ versus cell thickness

This section first derives a relation between $\bar{R}_z$ and the cell thickness L_{cell}, which was used to model the data of Fig. 4F. Subsequently, the experimental determination of $\bar{R}_z$ and L_{cell} from DC z-scan data is described.

The partial exclusion of fluorescently labeled protein from the cortex reduces the fluorescence area Λ^G as compared to the area Λ^G_{ref} corresponding to the hypothetical case of no exclusion. The ratio of these two areas is denoted as the axially averaged partition coefficient,

$$\bar{R}_z = \frac{\Lambda^G}{\Lambda^G_{\mathrm{ref}}} = \frac{A^G}{A^G_{\mathrm{ref}}} = \frac{L_{cyto} c_{cyto} + 2 L_C R_C c_{cyto}}{L_{cell} c_{cyto}}, \qquad \text{S1}$$

where the proportionality between Λ and A (Eq. 7) was used to express the ratio in terms of the integrated concentration areas. Since the cell thickness is defined by (Fig. 3B),

$$L_{cell} = L_{cyto} + L_C^T + L_C^B = L_{cyto} + 2L_C, \qquad \text{S2}$$

where $L_C = (L_C^T + L_C^B)/2$ defines the average cortex thickness, the axially averaged partition coefficient simplifies to

$$\bar{R}_z = 1 + (R_C - 1)\frac{2L_C}{L_{cell}} \qquad \text{S3}$$

This equation predicts $\bar{R}_z \to 1$ for very thick sections ($L_{cyto} \gg L_C$). As the cell thickness L_{cell} decreases, the value of $\bar{R}_z$ drops. It reaches a limiting value of $\bar{R}_z = R_C$ for very thin cell section composed entirely of cortex $L_{cell} = 2L_C$. Thus, DC z-scans in thin regions near

the cell perimeter combined with DC z-scans in thick regions of the same cells allow direct estimation of the partition coefficient.

While this relatively simple model sets a minimum cell thickness of $L_{cell} = 2L_C$, where L_C denotes the average cortical thickness in a given cell type, in practice very thin cellular regions were found where the realized cell thickness is less than this value, $L_{cell} < 2L_C$. This implies that in these regions of the cell the local cortex thickness is less than L_C. Assuming that these thin sections are composed entirely of cortex, Eq. S3 is generalized to,

$$\overline{R}_z = \begin{cases} R_C & \text{if} \quad L_{cell} < 2L_C \\ 1 + (R_C - 1)\dfrac{2L_C}{L_{cell}} & \text{if} \quad L_{cell} > 2L_C \end{cases}. \qquad \text{S4}$$

Experimental determination of $\overline{R}_z$ from DC z-scans in thin and thick regions of the cell is reflected in the decrease of the integrated fluorescence area Λ^G as a function of cell thickness (Eq. S1). To quantify the effect and utilize Eq. S1, an estimate was needed for the reference area $\Lambda^G_{ref} = L_{cell} g^G_{cyto}$, where $g^G_{cyto} = c^G_{cyto}\eta$ (Eqs. 7 and S1). The cell thickness L_{cell} was determined from the mCherry signal in the red detection channel as described in the next paragraph, while the amplitude g^G_{cyto} was estimated from a scan through a thick cell section (L_{cell} > 1.5 µm) where g^G_{cyto} can be reliably extracted from fitting the DC z-scan traces.

Determination of the thickness L_{cell} from DC z-scan data of a cell section that is thick with respect to the RIPSF width is obtained by a direct fit of the intensity profile from the mCherry signal to a slab model (Eq. 11) determines the cell thickness by L_{cell} =

$b^{CH} - a^{CH}$. For cell sections that are thin with respect to the RIPSF, cell thickness cannot be reliably determined from fitting to a slab model (*32*). However, because the fluorescent protein mCherry is distributed uniformly throughout the cell interior, its z-scan intensity provides a measure of local cell thickness L_{cell}. In this manner the cell thickness in thin sections can be determined from the integrated fluorescence Λ^{CH} of the intensity profile. Λ^{CH} is proportional to the integrated concentration area A^{CH} (Eq. 7), which is $L_{cell}c^{CH}$ for the slab geometry, where the mCherry concentration c^{CH} is assumed constant throughout the cell. Thus, the ratio of the integrated intensity profiles from a thick and a thin section measured in the same cell equals their thickness ratio, $\Lambda_{thin}^{CH} / \Lambda_{thick}^{CH} = L_{cell,thin} / L_{cell,thick}$, which was used to determine the thickness of thin cell sections. Although previous work (*32*) defined the cutoff between thick and thin cell lections as L_{cell} = 0.5 μm, a more conservative value of 1.5 μm was used in this study to improve the precision of length measurements.

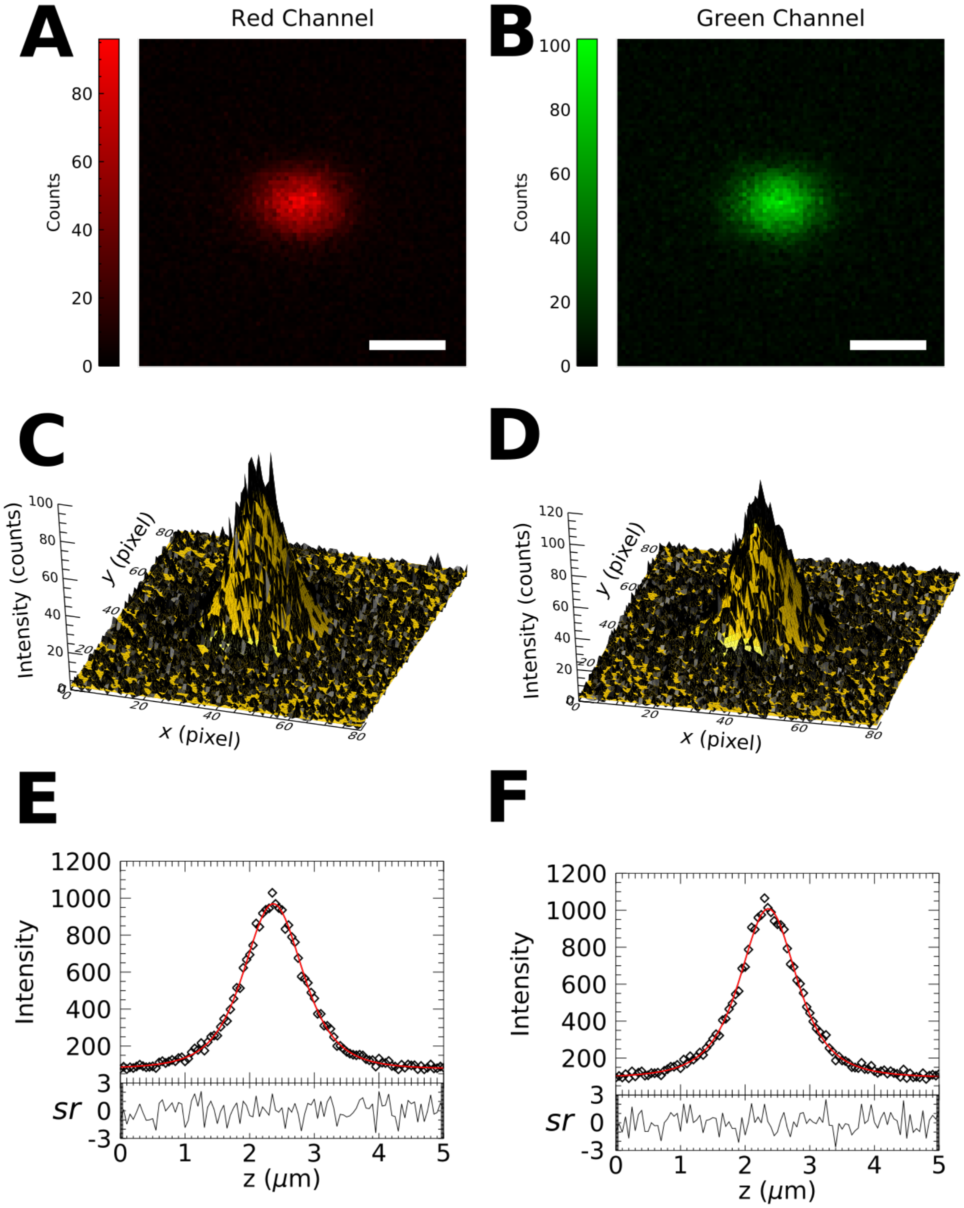

Figure S1: Absence of chromatic aberrations in the two-photon PSF. A z-stack of TetraSpeck beads was acquired on the same microscope used for DC z-scan data collection. (A) & (B) Images of a single 0.1 μm TetraSpeck bead in the red and green channels, respectively. Scale bar = 500 nm. Images represent the data after summing over the Z-dimension. (C) & (D) The intensity profiles from panels A & B are fit to 2D Gaussian functions to localize the bead in the XY-dimensions in both channels. The red and green localizations in X and Y differ by -1.5 ± 2.3 nm and 1.4 ± 1.7 nm, respectively. (E) & (F) The z-stack is summed over the XY dimensions to produce a radially integrated z-profile of the bead in the red and green channels, respectively (diamonds). These z-scan profiles are fit to δ layer models (red solid curve) to localize the bead in the vertical dimension in both channels. The red and green vertical localizations of the bead differ by 6.5 ± 6.5 nm.

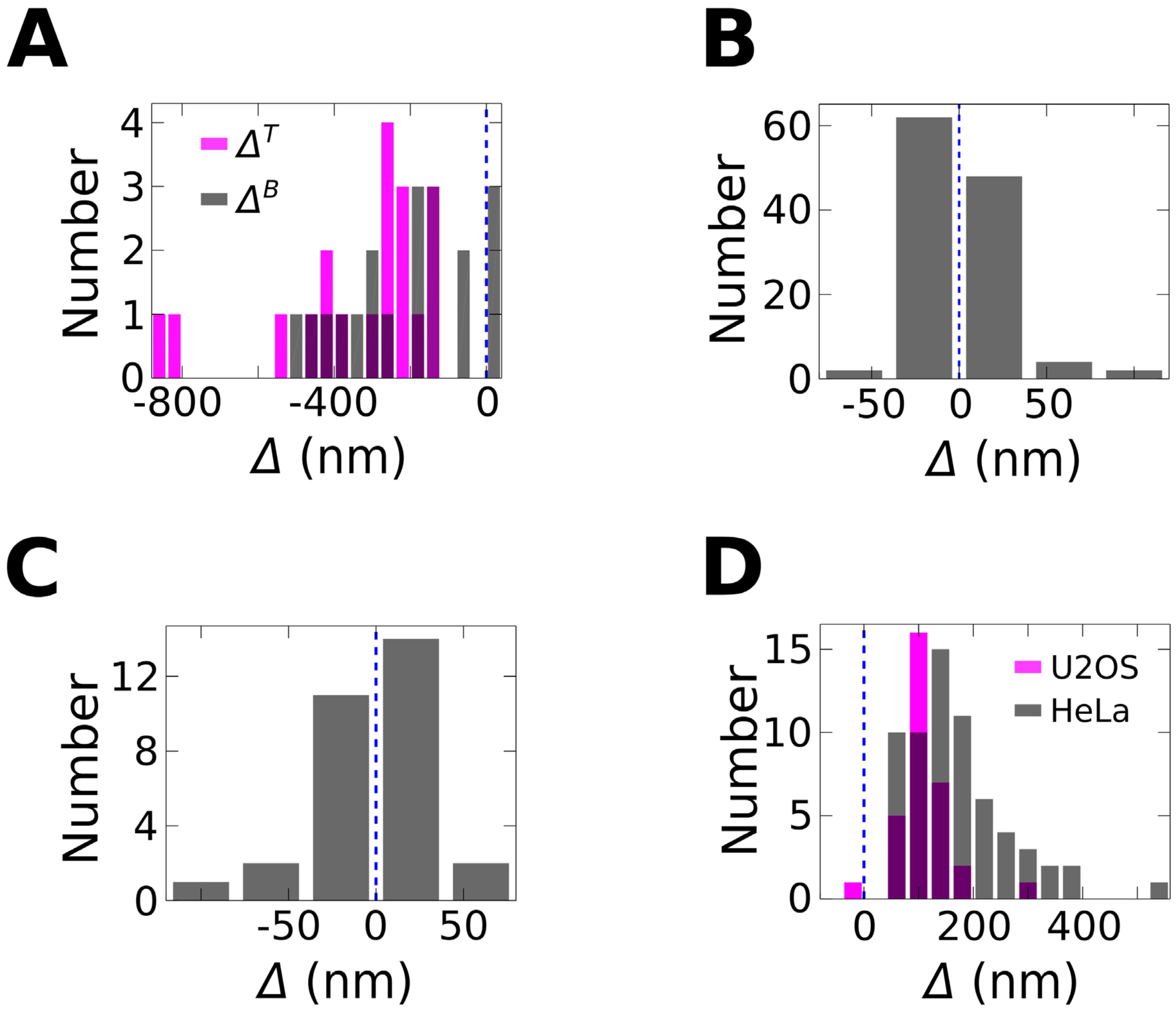

Fig. S2: Scaled Histograms of *Δ*. The histograms from Fig. 1 are reproduced with independently scaled horizontal axes. A) EGFP and mCherry-RXR, B) EGFP and mCherry, C) EGFP-HRas and mCherry, and D) A3G-EGFP and mCherry.

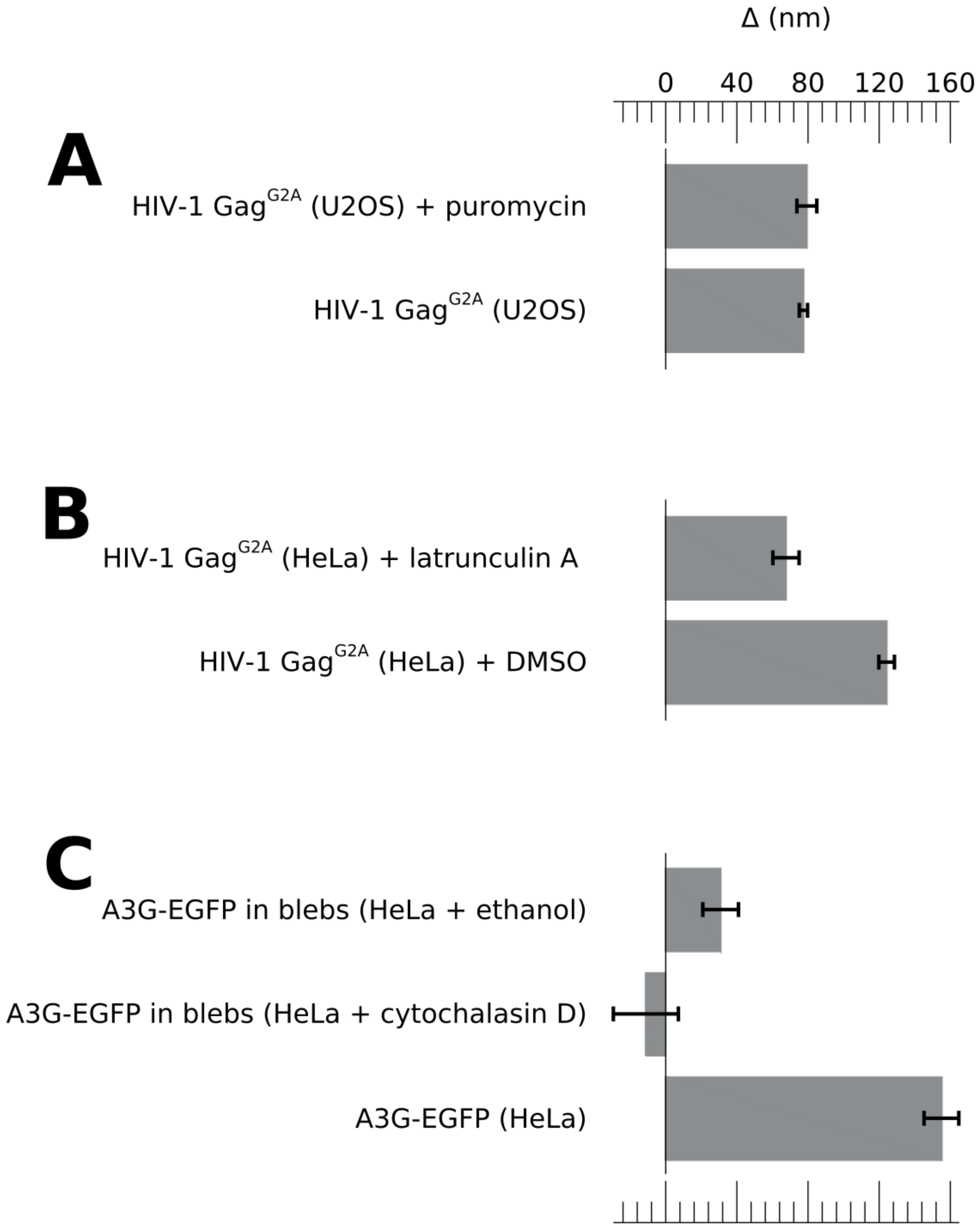

Figure S3: Depletion length in cells treated with puromycin and F-actin disrupting agents. A) DC z-scan measurements of HIV-1 Gag^{G2A} in the cytoplasm of U2OS cells treated with 100 µg/mL puromycin yielded the same Δ values as measurements performed in untreated cells. B) DC z-scan measurements in the cytoplasm of HeLa cells treated with 0.15 µg/mL latrunculin A showed significantly reduced Δ values compared to a solvent control. C) DC z-scan measurements of A3G-EGFP in cell associated blebs generated by treating HeLa cells with ethanol or cytochalasin D yielded much lower Δ values compared to A3G in the cytoplasm of untreated HeLa cells.

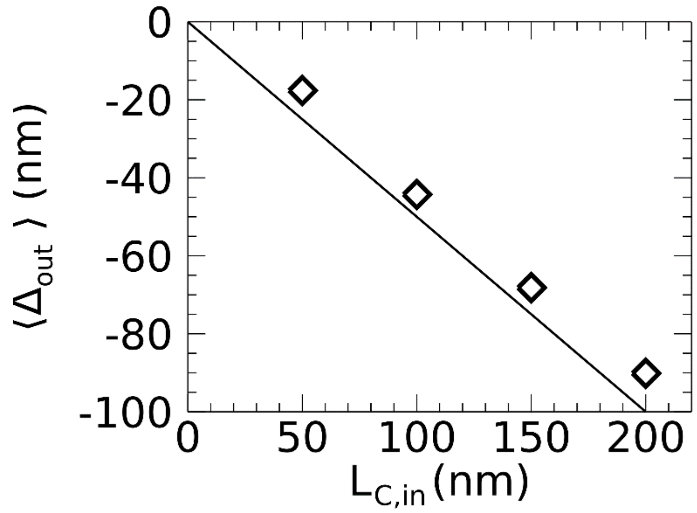

Fig. S4: Modeling of effective depletion length: The relation $|\Delta| = L_C / 2$ was tested with simulated traces (see Materials and Methods) of a SSS^{CH}- $\delta S\delta^G$ model, which were fit to a $\delta S\delta^{CH}$- $\delta S\delta^G$ model to extract the effective depletion length $<\Delta_{out}>$. This fitted length is plotted versus the cortex length $L_{C,in}$ chosen for the simulated SSS^{CH} model. Each data point represents the averaged Δ_{out} from simulations conducted over a range of fluorescent amplitude and L_{cell} parameter space that is similar to the range covered by DC z-scans measured in cells expressing EGFP-HRas and Lifeact-mApple. Additionally, $L_{cell} \geq 2.5$ µm and a membrane fraction $m \geq 0.5$ was chosen to minimize biases in the fitter estimates (20). The line represents the theoretical model, $\Delta_{out} = -L_C / 2$, which agrees with the simulated values to within 10 nm. This result demonstrates that the relation $|\Delta| = L_C / 2$ is experimentally accurate and produces negligible error.

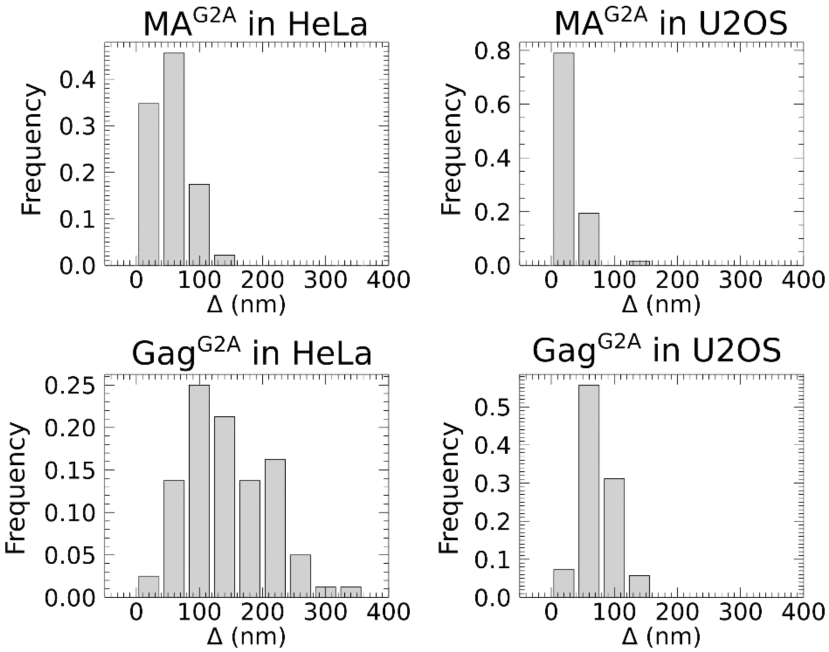

Fig. S5: Cell-line dependent depletion length. Histogram of effective depletion lengths measured for the indicated constructs in HeLa and U2OS cells.

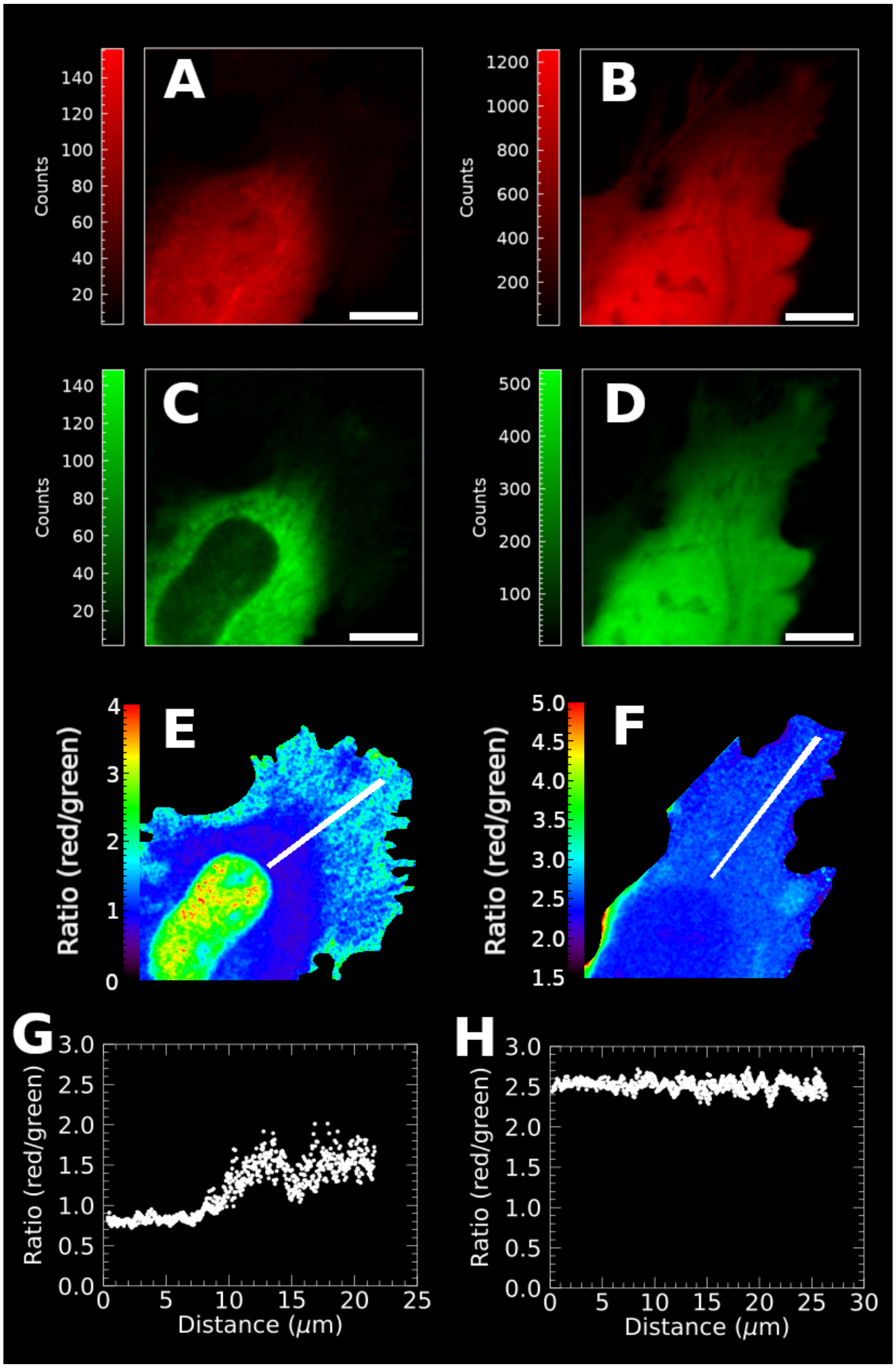

Figure S6: Two-photon ratio imaging qualitatively detects cortical partitioning in peripheral cytoplasm. The summed z-stack of the red and green detection channel of a HeLa cell co-expressing Gag^{G2A}-EGFP with mCherry (A, C) and EGFP with mCherry (B, D). The red channel (A, B), green channel (C, D), and red to green channel ratio (E, F) images are shown. Line plots of the red/green ratio along the white line (E, F) are shown for the Gag^{G2A}-EGFP (G) and EGFP (H) cell. Zero distance represents the point within each stripe that is closest to the cell nucleus. The red/green ratio increases near the periphery of the cell expressing Gag^{G2A}-EGFP, indicating the relative exclusion of Gag^{G2A}-EGFP from these regions. In contrast, the red/green ratio in the cell expressing EGFP is approximately constant. Scale bars = 10 μm.

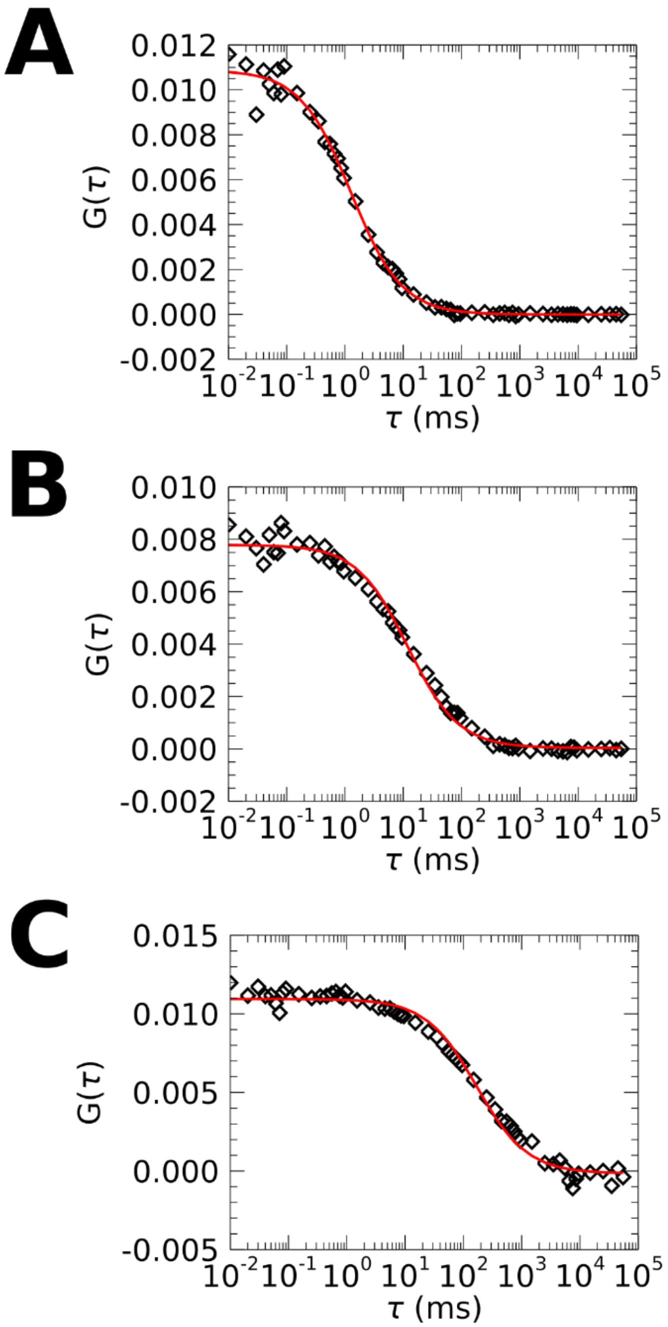

Figure S7: Representative autocorrelation functions. Representative autocorrelation functions (ACFs) measured for (A) EGFP, (B) HIV-1 Gag^{G2A}-EGFP, and (C) A3G-EGFP in HeLa cells are plotted as black diamonds. A fit to a 2D Gaussian diffusion model is plotted as the red curves. Note that both HIV-1 Gag^{G2A} -EGFP and A3G-EGFP show a slightly broadened decay of the autocorrelation curve, which may indicate heterogeneity in the diffusion coefficients within the sample, anomalous diffusion, or both *(81)*. The characteristic diffusion time τ_D corresponds to the time where the ACF reached half its initial amplitude, which is adequately determined by the simple diffusion model.

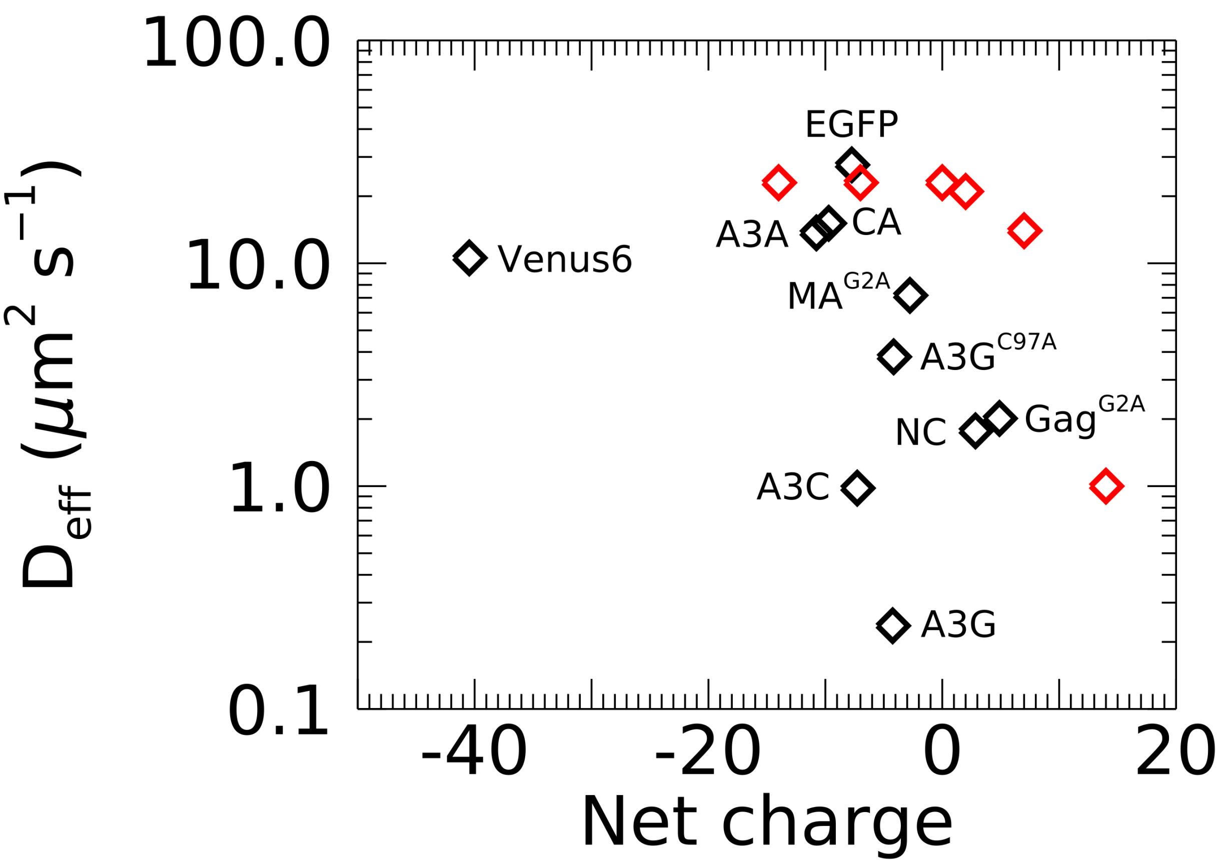

Fig. S8: Effective diffusion coefficient versus protein net charge (black diamonds). The red diamonds correspond to diffusion coefficients estimated in *(49)* for fast-diffusing regions of cytoplasm. Net charges were calculated from the amino acid sequence of each protein using an average residue charge at pH 7.4 as described in *(54)*.

Sample name	STDEV across all z-scans (nm)	average STDEV within single cells (nm)
EGFP	20	13
HIV-1 Gag^{G2A}-EGFP	66	54
A3G-EGFP	93	71
A3G^{C97A}-EGFP	39	32
A3A-EGFP	15	12
A3C-EGFP	44	37
SYTO RNASelect	141	73
MA^{G2A}	28	17
Venus$_6$	36	20
GABARAP-EGFP	25	19

Table S1: Variability in measured depletion values. The standard deviation of the depletion length Δ calculated across all z-scans in each dataset is compared to the average standard deviation of the depletion length at distinct locations within the same cell. All values were obtained from HeLa cells. The majority of the variability is accounted for by intracellular heterogeneity.

	r_{eff} (nm)
**Gag^{G2A}**	15.8
**A3G**	37.1
**A3G^{C97A}**	10.6
**A3A**	2.4
**A3C**	22.4
**SytoRNASelect**	19.2
**NC**	16.9
**CA**	1.9
**MA^{G2A}**	6.1
**Venus$_6$**	3.8
**GABARAP**	5.7

Table S2: Effective hydrodynamic radii derived from the hydrodynamic scaling model in HeLa cells. Eq. 3 was solved for r_{eff} and then evaluated with the model parameter values used in the text and the effective diffusion coefficient D_{eff} measured for each protein by FCS.

REFERENCES AND NOTES

1. P. Chugh, E. K. Paluch, The actin cortex at a glance. *J. Cell Sci.* **131**, jcs186254 (2018).

2. A. Kusumi, T. K. Fujiwara, R. Chadda, M. Xie, T. A. Tsunoyama, Z. Kalay, R. S. Kasai, K. G. N. Suzuki, Dynamic organizing principles of the plasma membrane that regulate signal transduction: Commemorating the fortieth anniversary of Singer and Nicolson's fluid-mosaic model. *Annu. Rev. Cell Dev. Biol.* **28**, 215–250 (2012).

3. M. D. Blower, Molecular insights into intracellular RNA localization. *Int. Rev. Cell Mol. Biol.* **302**, 1–39 (2013).

4. C. Medioni, K. Mowry, F. Besse, Principles and roles of mRNA localization in animal development. *Development* **139**, 3263–3276 (2012).

5. E. Lécuyer, H. Yoshida, N. Parthasarathy, C. Alm, T. Babak, T. Cerovina, T. R. Hughes, P. Tomancak, H. M. Krause, Global analysis of mRNA localization reveals a prominent role in organizing cellular architecture and function. *Cell* **131**, 174–187 (2007).

6. A. Gopal, Z. H. Zhou, C. M. Knobler, W. M. Gelbart, Visualizing large RNA molecules in solution. *RNA* **18**, 284–299 (2012).

7. A. Borodavka, S. W. Singaram, P. G. Stockley, W. M. Gelbart, A. Ben-Shaul, R. Tuma, Sizes of long RNA molecules are determined by the branching patterns of their secondary structures. *Biophys. J.* **111**, 2077–2085 (2016).

8. S. S. Sommer, J. E. Cohen, The size distributions of proteins, mRNA, and nuclear RNA. *J. Mol. Evol.* **15**, 37–57 (1980).

9. I. Legnini, J. Alles, N. Karaiskos, S. Ayoub, N. Rajewsky, FLAM-seq: Full-length mRNA sequencing reveals principles of poly(A) tail length control. *Nat. Methods* **16**, 879–886 (2019).

10. M. F. Tam, J. A. Dodd, W. E. Hill, Physical characteristics of 16 S rRNA under reconstitution conditions. *J. Biol. Chem.* **256**, 6430–6434 (1981).

11. A. Verschoor, J. Frank, Three-dimensional structure of the mammalian cytoplasmic ribosome. *J. Mol. Biol.* **214**, 737–749 (1990).

12. G. T. Charras, T. J. Mitchison, L. Mahadevan, Animal cell hydraulics. *J. Cell Sci.* **122**, 3233–3241 (2009).

13. E. Moeendarbary, L. Valon, M. Fritzsche, A. R. Harris, D. A. Moulding, A. J. Thrasher, E. Stride, L. Mahadevan, G. T. Charras, The cytoplasm of living cells behaves as a poroelastic material. *Nat. Mater.* **12**, 253–261 (2013).

14. K. Luby-Phelps, P. E. Castle, D. L. Taylor, F. Lanni, Hindered diffusion of inert tracer particles in the cytoplasm of mouse 3T3 cells. *Proc. Natl. Acad. Sci. U.S.A.* **84**, 4910–4913 (1987).

15. K. Luby-Phelps, D. L. Taylor, Subcellular compartmentalization by local differentiation of cytoplasmic structure. *Cell Motil. Cytoskeleton* **10**, 28–37 (1988).

16. L. Hou, F. Lanni, K. Luby-Phelps, Tracer diffusion in F-actin and Ficoll mixtures. Toward a model for cytoplasm. *Biophys. J.* **58**, 31–43 (1990).

17. G. L. Lukacs, P. Haggie, O. Seksek, D. Lechardeur, N. Freedman, A. S. Verkman, Size-dependent DNA mobility in cytoplasm and nucleus. *J. Biol. Chem.* **275**, 1625–1629 (2000).

18. E. Dauty, A. S. Verkman, Actin cytoskeleton as the principal determinant of size-dependent DNA mobility in cytoplasm: A new barrier for non-viral gene delivery. *J. Biol. Chem.* **280**, 7823–7828 (2005).

19. K. Luby-Phelps, Effect of cytoarchitecture on the transport and localization of protein synthetic machinery. *J. Cell. Biochem.* **52**, 140–147 (1993).

20. I. Angert, S. R. Karuka, J. Hennen, Y. Chen, J. P. Albanesi, L. M. Mansky, J. D. Mueller, Sensitive detection of protein binding to the plasma membrane with dual-color Z-scan fluorescence. *Biophys. J.* **118**, 281–293 (2020).

21. E. M. Smith, P. J. Macdonald, Y. Chen, J. D. Mueller, Quantifying protein-protein interactions of peripheral membrane proteins by fluorescence brightness analysis. *Biophys. J.* **107**, 66–75 (2014).

22. E. M. Smith, J. Hennen, Y. Chen, J. D. Mueller, Z-scan fluorescence profile deconvolution of cytosolic and membrane-associated protein populations. *Anal. Biochem.* **480**, 11–20 (2015).

23. Y. N. Friew, V. Boyko, W.-S. Hu, V. K. Pathak, Intracellular interactions between APOBEC3G, RNA, and HIV-1 Gag: APOBEC3G multimerization is dependent on its association with RNA. *Retrovirology* **6**, 56 (2009).

24. Y. Iwatani, H. Takeuchi, K. Strebel, J. G. Levin, Biochemical activities of highly purified, catalytically active human APOBEC3G: Correlation with antiviral effect. *J. Virol.* **80**, 5992–6002 (2006).

25. S. Saha, I.-H. Lee, A. Polley, J. T. Groves, M. Rao, S. Mayor, Diffusion of GPI-anchored proteins is influenced by the activity of dynamic cortical actin. *Mol. Biol. Cell* **26**, 4033–4045 (2015).

26. T. Baumgart, A. T. Hammond, P. Sengupta, S. T. Hess, D. A. Holowka, B. A. Baird, W. W. Webb, Large-scale fluid/fluid phase separation of proteins and lipids in giant plasma membrane vesicles. *Proc. Natl. Acad. Sci. U.S.A.* **104**, 3165–3170 (2007).

27. F. Schneider, D. Waithe, M. P. Clausen, S. Galiani, T. Koller, G. Ozhan, C. Eggeling, E. Sezgin, Diffusion of lipids and GPI-anchored proteins in actin-free plasma membrane vesicles measured by STED-FCS. *Mol. Biol. Cell* **28**, 1507–1518 (2017).

28. T. M. Svitkina, Actin cell cortex: Structure and molecular organization. *Trends Cell Biol.* **30**, 556–565 (2020).

29. N. Morone, T. Fujiwara, K. Murase, R. S. Kasai, H. Ike, S. Yuasa, J. Usukura, A. Kusumi, Three-dimensional reconstruction of the membrane skeleton at the plasma membrane interface by electron tomography. *J. Cell Biol.* **174**, 851–862 (2006).

30. P. Chugh, A. G. Clark, M. B. Smith, D. A. D. Cassani, K. Dierkes, A. Ragab, P. P. Roux, G. Charras, G. Salbreux, E. K. Paluch, Actin cortex architecture regulates cell surface tension. *Nat. Cell Biol.* **19**, 689–697 (2017).

31. A. G. Clark, K. Dierkes, E. K. Paluch, Monitoring actin cortex thickness in live cells. *Biophys. J.* **105**, 570–580 (2013).

32. J. Riedl, A. H. Crevenna, K. Kessenbrock, J. H. Yu, D. Neukirchen, M. Bista, F. Bradke, D. Jenne, T. A. Holak, Z. Werb, M. Sixt, R. Wedlich-Soldner, Lifeact: A versatile marker to visualize F-actin. *Nat. Methods* **5**, 605–607 (2008).

33. P. J. Macdonald, Y. Chen, X. Wang, Y. Chen, J. D. Mueller, Brightness analysis by Z-scan fluorescence fluctuation spectroscopy for the study of protein interactions within living cells. *Biophys. J.* **99**, 979–988 (2010).

34. J. G. Levin, J. Guo, I. Rouzina, K. Musier-Forsyth, Nucleic acid chaperone activity of HIV-1 nucleocapsid protein: Critical role in reverse transcription and molecular mechanism. *Prog. Nucleic Acid Res. Mol. Biol.* **80**, 217–286 (2005).

35. R. N. De Guzman, Z. R. Wu, C. C. Stalling, L. Pappalardo, P. N. Borer, M. F. Summers, Structure of the HIV-1 nucleocapsid protein bound to the SL3 psi-RNA recognition element. *Science* **279**, 384–388 (1998).

36. A. Rein, Nucleic acid chaperone activity of retroviral Gag proteins. *RNA Biol.* **7**, 700–705 (2010).

37. V. Chukkapalli, S. J. Oh, A. Ono, Opposing mechanisms involving RNA and lipids regulate HIV-1 Gag membrane binding through the highly basic region of the matrix domain. *Proc. Natl. Acad. Sci. U.S.A.* **107**, 1600–1605 (2010).

38. S. B. Kutluay, T. Zang, D. Blanco-Melo, C. Powell, D. Jannain, M. Errando, P. D. Bieniasz, Global changes in the RNA binding specificity of HIV-1 gag regulate virion genesis. *Cell* **159**, 1096–1109 (2014).

39. J. Li, Y. Chen, M. Li, M. A. Carpenter, R. M. McDougle, E. M. Luengas, P. J. Macdonald, R. S. Harris, J. D. Mueller, APOBEC3 multimerization correlates with HIV-1 packaging and restriction activity in living cells. *J. Mol. Biol.* **426**, 1296–1307 (2014).

40. K. Luby-Phelps, D. L. Taylor, F. Lanni, Probing the structure of cytoplasm. *J. Cell Biol.* **102**, 2015–2022 (1986).

41. B. R. Cullen, Role and mechanism of action of the APOBEC3 family of antiretroviral resistance factors. *J. Virol.* **80**, 1067–1076 (2006).

42. J. S. Albin, R. S. Harris, Interactions of host APOBEC3 restriction factors with HIV-1 in vivo: Implications for therapeutics. *Expert Rev. Mol. Med.* **12**, e4 (2010).

43. J. F. Hultquist, J. A. Lengyel, E. W. Refsland, R. S. LaRue, L. Lackey, W. L. Brown, R. S. Harris, Human and rhesus APOBEC3D, APOBEC3F, APOBEC3G, and APOBEC3H demonstrate a conserved capacity to restrict Vif-deficient HIV-1. *J. Virol.* **85**, 11220–11234 (2011).

44. L. W. Janson, K. Ragsdale, K. Luby-Phelps, Mechanism and size cutoff for steric exclusion from actin-rich cytoplasmic domains. *Biophys. J.* **71**, 1228–1234 (1996).

45. A. P. Minton, Confinement as a determinant of macromolecular structure and reactivity. *Biophys. J.* **63**, 1090–1100 (1992).

46. J. C. Giddings, E. Kucera, C. P. Russell, M. N. Myers, Statistical theory for the equilibrium distribution of rigid molecules in inert porous networks. Exclusion chromatography. *J. Phys. Chem.* **72**, 4397–4408 (1968).

47. T. Kalwarczyk, N. Ziebacz, A. Bielejewska, E. Zaboklicka, K. Koynov, J. Szymański, A. Wilk, A. Patkowski, J. Gapiński, H.-J. Butt, R. Hołyst, Comparative analysis of viscosity of complex liquids and cytoplasm of mammalian cells at the nanoscale. *Nano Lett.* **11**, 2157–2163 (2011).

48. R. Holyst, A. Bielejewska, J. Szymański, A. Wilk, A. Patkowski, J. Gapiński, A. Zywociński, T. Kalwarczyk, E. Kalwarczyk, M. Tabaka, N. Ziebacz, S. A. Wieczorek, Scaling form of viscosity at

all length-scales in poly(ethylene glycol) solutions studied by fluorescence correlation spectroscopy and capillary electrophoresis. *Phys. Chem. Chem. Phys.* **11**, 9025–9032 (2009).

49. L. Xiang, K. Chen, R. Yan, W. Li, K. Xu, Single-molecule displacement mapping unveils nanoscale heterogeneities in intracellular diffusivity. *Nat. Methods* **17**, 524–530 (2020).

50. T. P. Stossel, P. A. Janmey, K. S. Zaner, in *Cytomechanics: The Mechanical Basis of Cell Form and Structure*, J. Bereiter-Hahn, O. R. Anderson, W.-E. Reif, Eds. (Springer, 1987), pp. 131–153.

51. J.-F. Joanny, J. Prost, Active gels as a description of the actin-myosin cytoskeleton. *HFSP J.* **3**, 94–104 (2009).

52. T. C. Laurent, J. Killander, A theory of gel filtration and its exeperimental verification. *J. Chromatogr. A* **14**, 317–330 (1964).

53. K. C. Duong-Ly, S. B. Gabelli, Gel filtration chromatography (size exclusion chromatography) of proteins. *Methods Enzymol.* **541**, 105–114 (2014).

54. T. Kaur, I. Alshareedah, W. Wang, J. Ngo, M. M. Moosa, P. R. Banerjee, Molecular crowding tunes material states of ribonucleoprotein condensates. *Biomolecules* **9**, 71 (2019).

55. A. A. M. André, E. Spruijt, Liquid-liquid phase separation in crowded environments. *Int. J. Mol. Sci.* **21**, 5908 (2020).

56. D. W. Provance, A. McDowall, M. Marko, K. Luby-Phelps, Cytoarchitecture of size-excluding compartments in living cells. *J. Cell Sci.* **106**, 565–577 (1993).

57. R. Kumar, S. Saha, B. Sinha, Cell spread area and traction forces determine myosin-II-based cortex thickness regulation. *Biochim. Biophys. Acta Mol. Cell Res.* **1866**, 118516 (2019).

58. T. M. Svitkina, A. A. Shevelev, A. D. Bershadsky, V. I. Gelfand, Cytoskeleton of mouse embryo fibroblasts. Electron microscopy of platinum replicas. *Eur. J. Cell Biol.* **34**, 64–74 (1984).

59. G. Blobel, D. Sabatini, Dissociation of mammalian polyribosomes into subunits by puromycin. *Proc. Natl. Acad. Sci. U.S.A.* **68**, 390–394 (1971).

60. M. Delarue, G. P. Brittingham, S. Pfeffer, I. V. Surovtsev, S. Pinglay, K. J. Kennedy, M. Schaffer, J. I. Gutierrez, D. Sang, G. Poterewicz, J. K. Chung, J. M. Plitzko, J. T. Groves, C. Jacobs-Wagner, B. D. Engel, L. J. Holt, mTORC1 controls phase separation and the biophysical properties of the cytoplasm by tuning crowding. *Cell* **174**, 338–349.e20 (2018).

61. K. Kwapiszewska, K. Szczepański, T. Kalwarczyk, B. Michalska, P. Patalas-Krawczyk, J. Szymański, T. Andryszewski, M. Iwan, J. Duszyński, R. Hołyst, Nanoscale viscosity of cytoplasm is conserved in human cell lines. *J. Phys. Chem. Lett.* **11**, 6914–6920 (2020).

62. J. Chen, D. Grunwald, L. Sardo, A. Galli, S. Plisov, O. A. Nikolaitchik, D. Chen, S. Lockett, D. R. Larson, V. K. Pathak, W.-S. Hu, Cytoplasmic HIV-1 RNA is mainly transported by diffusion in the presence or absence of Gag protein. *Proc. Natl. Acad. Sci. U.S.A.* **111**, E5205–E5213 (2014).

63. J. J. Blum, G. Lawler, M. Reed, I. Shin, Effect of cytoskeletal geometry on intracellular diffusion. *Biophys. J.* **56**, 995–1005 (1989).

64. K. Luby-Phelps, F. Lanni, D. L. Taylor, The submicroscopic properties of cytoplasm as a determinant of cellular function. *Annu. Rev. Biophys. Biophys. Chem.* **17**, 369–396 (1988).

65. D. S. Banks, C. Fradin, Anomalous diffusion of proteins due to molecular crowding. *Biophys. J.* **89**, 2960–2971 (2005).

66. A. Miermont, F. Waharte, S. Hu, M. N. McClean, S. Bottani, S. Léon, P. Hersen, Severe osmotic compression triggers a slowdown of intracellular signaling, which can be explained by molecular crowding. *Proc. Natl. Acad. Sci. U.S.A.* **110**, 5725–5730 (2013).

67. M. Weiss, M. Elsner, F. Kartberg, T. Nilsson, Anomalous subdiffusion is a measure for cytoplasmic crowding in living cells. *Biophys. J.* **87**, 3518–3524 (2004).

68. C. I. Lacayo, J. A. Theriot, Listeria monocytogenes actin-based motility varies depending on subcellular location: A kinematic probe for cytoarchitecture. *Mol. Biol. Cell* **15**, 2164–2175 (2004).

69. J.-Y. Youn, B. J. A. Dyakov, J. Zhang, J. D. R. Knight, R. M. Vernon, J. D. Forman-Kay, A.-C. Gingras, Properties of stress granule and p-body proteomes. *Mol. Cell* **76**, 286–294 (2019).

70. A. A. Hyman, C. A. Weber, F. Jülicher, Liquid-liquid phase separation in biology. *Annu. Rev. Cell Dev. Biol.* **30**, 39–58 (2014).

71. L.-P. Bergeron-Sandoval, N. Safaee, S. W. Michnick, Mechanisms and consequences of macromolecular phase separation. *Cell* **165**, 1067–1079 (2016).

72. R. Wollman, T. Meyer, Coordinated oscillations in cortical actin and $Ca2^{+}$ correlate with cycles of vesicle secretion. *Nat. Cell Biol.* **14**, 1261–1269 (2012).

73. P. Li, A. T. Bademosi, J. Luo, F. A. Meunier, Actin remodeling in regulated exocytosis: Toward a mesoscopic view. *Trends Cell Biol.* **28**, 685–697 (2018).

74. E. D. Olson, K. Musier-Forsyth, Retroviral Gag protein-RNA interactions: Implications for specific genomic RNA packaging and virion assembly. *Semin. Cell Dev. Biol.* **86**, 129–139 (2019).

75. A. Derdowski, L. Ding, P. Spearman, A novel fluorescence resonance energy transfer assay demonstrates that the human immunodeficiency virus type 1 Pr55Gag I domain mediates Gag-Gag interactions. *J. Virol.* **78**, 1230–1242 (2004).

76. C. Thaler, S. V. Koushik, H. L. Puhl, P. S. Blank, S. S. Vogel, Structural rearrangement of CaMKIIα catalytic domains encodes activation. *Proc. Natl. Acad. Sci. U.S.A.* **106**, 6369–6374 (2009).

77. B. Wu, Y. Chen, J. D. Müller, Fluorescence fluctuation spectroscopy of mCherry in living cells. *Biophys. J.* **96**, 2391–2404 (2009).

78. B. Wu, Y. Chen, J. D. Müller, Heterospecies partition analysis reveals binding curve and stoichiometry of protein interactions in living cells. *Proc. Natl. Acad. Sci. U.S.A.* **107**, 4117–4122 (2010).

79. Y. Chen, H.-Q. Sun, J. P. Eichorst, J. P. Albanesi, H. Yin, J. D. Mueller, Comobility of GABARAP and phosphatidylinositol 4-kinase 2A on cytoplasmic vesicles. *Biochemistry* **57**, 3556–3559 (2018).

80. J. D. Müller, Y. Chen, E. Gratton, [4] Fluorescence correlation spectroscopy. *Methods Enzymol.* **361**, 69–92 (2003).

81. T. Kalwarczyk, K. Kwapiszewska, K. Szczepanski, K. Sozanski, J. Szymanski, B. Michalska, P. Patalas-Krawczyk, J. Duszynski, R. Holyst, Apparent anomalous diffusion in the cytoplasm of human cells: The effect of probes' polydispersity. *J. Phys. Chem. B* **121**, 9831–9837 (2017).
